# Feasibility of Music-Assisted Treadmill Training in Parkinson's Disease Patients With and Without Deep Brain Stimulation: Insights From an Ongoing Pilot Randomized Controlled Trial

**DOI:** 10.3389/fneur.2020.00790

**Published:** 2020-09-04

**Authors:** Mareike Gooßes, Jochen Saliger, Ann-Kristin Folkerts, Jörn Nielsen, Jürgen Zierer, Paula Schmoll, Annika Niepold, Liz Colbach, Janna Leemhuis, Lea Engels, Maria van Krüchten, Anja Ophey, Niels Allert, Hans Karbe, Elke Kalbe

**Affiliations:** ^1^Medical Psychology | Neuropsychology and Gender Studies, Faculty of Medicine and University Hospital Cologne, University of Cologne, Cologne, Germany; ^2^Neurological Rehabilitation Center Godeshoehe, Bonn, Germany

**Keywords:** Parkinson's disease, deep brain stimulation, music assisted treadmill, non-pharmacological treatment, feasibility, randomized controlled trial

## Abstract

**Background:** Music-assisted treadmill training (MATT) is a new therapeutic approach for Parkinson's disease (PD) patients, combining treadmill training with rhythmic auditory cueing and visual feedback. PD studies have shown larger positive effects on motor outcomes than usual treadmill training. However, effects on cognition, in contrast, are less clear. Existing studies provided intensive training protocols and included only stable medicated patients. Thus, a pilot randomized controlled trial was designed to analyze the feasibility of a shorter training protocol as well as preliminary effects on cognition, motor function, and patient-centered outcomes in a rehabilitation setting where PD patients with and without deep brain stimulation (DBS) undergo adaptation of medication and DBS settings. Here, we present the results from the feasibility analysis of the still ongoing trial.

**Methods:** Non-demented PD patients with and without DBS were recruited during their inpatient rehabilitation and randomized to an experimental group (EG; 20 min MATT) or an active control group (CG; 20 min bike ergometer training). The trainings took place for 8 consecutive days and were added to the usual rehabilitation. Feasibility was assessed with the following parameters: patients' study protocol acceptance, study protocol transferability into clinical routine, training-induced adverse events, and patients' training perception.

**Results:** Thirty-two patients (EG: *n* = 15; CG: *n* = 17; 72% DBS) were included. The study protocol was well-accepted (inclusion rate: 84%). It was transferable into clinical routines; dropout rates of 40% (EG) and 18% (CG) were observed. However, an in-depth analysis of the dropout cohort did not reveal intervention-related dropout reasons. The MATT and the standard ergometer training showed no adverse events and were positively perceived by PD patients with and without DBS.

**Conclusion:** MATT was shown to be a feasible, safe, and enjoyable treatment option in PD patients with and without DBS. Furthermore, the dropout cohort analysis revealed some exciting first insights into possible dropout reasons that go beyond the form of intervention. Therefore, research would benefit from a common practice of dropout analyses, as this would enhance our understanding of patients' therapy adherence and expectations.

## Introduction

Parkinson's disease (PD) is defined by the motor symptoms akinesia/bradykinesia, rigidity, resting tremor, and postural instability (1). However, growing evidence exists that PD is a more complex, multidimensional disease that goes along with motor symptoms as well as several non-motor symptoms (2–5).

Among the motor symptoms, the alterations in the ability to move caused by a loss of automaticity, rhythmicity, velocity, and effectiveness are clinically the most prominent features of the disease (6–11). These symptoms become especially evident in patients' gait and balance performance, which are usually impaired causing slowness/fastening of gait, higher step variability, reduced walking dynamics, and/or freezing of gait (11–14) and are likely to increase patients' inactivity (15) and disease burden (16, 17).

Pathophysiologically, these changes in movement and especially in gait are due to alterations in networks involving the basal ganglia and other brain structures (6, 7, 10, 18). These alterations are suggested to be also responsible for some of the non-motor symptoms that occur in PD (6, 8, 10). Among these, especially the cognitive functions are strongly discussed to be impacted by the neuronal changes caused by the transmitter depletion (6, 8, 19). The changes in cognition, which are common in PD (20, 21), additionally decrease quality of life and increase disease burden (16, 17).

Some of the disease-related symptoms can be treated pharmacologically (1, 22) or by deep brain stimulation [DBS; (23, 24)], whereas other symptoms respond only limitedly, negatively, or not at all to PD medication (3, 6, 10, 25) and/or DBS (23, 26–28). Furthermore, for the treatment of cognitive dysfunction, only limited pharmacologic options exist (6, 29, 30). Therefore, non-pharmacologic therapies, such as neurorehabilitation, gain increasing interest to improve PD symptoms (27, 31–34).

Neurorehabilitation is a multi-professional approach aiming at restoration, compensation, and/or prevention of ability loss through training (31, 32). Physiotherapy is a major pillar of PD neurorehabilitation, as PD was longtime considered solely as a motor disorder (33, 35, 36). One main goal of physiotherapy is to preserve and improve automaticity, rhythmicity, velocity, and effectiveness of movement through different trainings (34, 37). For the gait and balance deficits commonly observed in PD, treadmill training is a widely used training tool (38, 39). Nevertheless, conventional treadmill training oftentimes targets only motor symptoms in a multidimensional disease. However, treadmill training has cognitive aspects when conducting the training itself (40), and few studies have also examined the positive effects of endurance training (e.g., treadmill training) on cognitive functioning (41).

Knowledge has increased that motor function and cognition are interrelated in PD and should be considered as linked entities in therapy (6, 27, 31, 42), especially during a cognitive (re-)learning process (6, 31, 42). Neurorehabilitation usually is such a cognitive skill–(re-)learning process, indicating that its success depends on available and accessible cognitive resources (6, 40, 43).

Furthermore, in PD, the loss of automaticity, rhythmicity, velocity, and effectiveness of actions requires that patients have to cognitively control them (6, 11, 44, 45). This results in an increased cognitive load, an increased risk for errors/failure, and fatigue (11, 45). Additionally, the cognitive impairment itself, which can even occur in PD patients before or at the time of the diagnosis (3, 21), might influence movement planning and execution (11, 45, 46), all together limiting patients' (re-) learning potential. However, several studies have shown that (re-)learning is possible in PD (39, 42, 47–50). Approaches that consider patients' limitations and help to bypass the deficient networks seem to be especially promising (51–53).

One of these approaches is rhythmic auditory cueing (RAC). RAC eases the initiation and continuation of repetitive sequential movements by providing an external temporal auditory stimulus [e.g., metronome, music, clapping; (54)]. Other studies have also used ecological sounds as cues (e.g., footsteps), which have proven to be effective, especially for spatiotemporal parameters of gait (55, 56). This is believed to help patients to overcome the automaticity and rhythmicity deficits (44, 57, 58), resulting in more fluent movement and reduced cognitive engagement (44, 58). RAC is often combined with gait training and has proven to be an effective therapy tool to improve different aspects of gait, such as walking speed, step length, and step frequency (52, 59, 60). Besides its previously mentioned effects on automaticity and rhythmicity, RAC gait training in combination with visual feedback is considered as a dual-task cognitive–motor training (61, 62), as it demands patients' attention to consciously step synchronic to the auditory cue and include the visual feedback in the next step, which are two concurrent tasks. This allows therapists and patients to take advantage of a cognitive–motor strategy bypassing deficient neuronal circuits (57, 63, 64). Studies showed that RAC also affects cognition (65, 66) and motor relearning (49, 50).

The relative new therapy device Gait Trainer 3.1 by Biodex offers a treadmill with integrated RAC and real-time visual feedback extending common treadmill training by cognitive–motor strategies. Studies on this device have shown superior effects of music-assisted treadmill training (MATT) on patients' gait (67–69), locomotion networks (67), and quality of life (69) compared with those of traditional treadmill training without RAC. However, these studies were conducted in special rehabilitation settings with high training intensities and duration and did not examine the effects on cognition (64, 67–69). Furthermore, patients' medication had to be unchanged before and during study inclusion (64, 67, 69). Although this methodological procedure has some advantages regarding the interpretability of changes, it might not represent “real-world” PD patients that are referred to inpatient rehabilitation with the indication also to optimize medication and/or DBS settings. The Godeshoehe rehabilitation clinic (Bonn, Germany) is specialized in post-surgery DBS programming and medication optimization so that PD patients characteristically undergo adjustment of medication and stimulation parameters during their inpatient rehabilitation (70, 71). It is, therefore, especially interesting for such a rehabilitation setting, if a study protocol like the present and the newly herein planned MATT is feasible and safe compared with the usual treatments. Moreover, this question is especially relevant to extend non-pharmacological therapy options in the PD-DBS cohort as literature on the (physical) trainability of those patients is scarce (72).

Therefore, we designed a pilot randomized controlled trial (RCT) with a 6-week follow-up evaluating the feasibility of our study protocol and study interventions as well as preliminary effects on cognition, motor function, and patient-centered outcomes. In this present article, we show the feasibility analysis of this ongoing pilot RCT, whereas the results on cognition, motor function, and patient-centered outcomes immediately after training and 6 weeks later will be reported after the recruitment is completed.

Regarding the feasibility of the study protocol, it was hypothesized that (i) PD inpatients at the rehabilitation clinic are interested in joining an additional 20 min of training either on the MATT or the ergometer; (ii) the study protocol can be transferred into clinical routines. Regarding the training feasibility, we expected that (iii) MATT will not cause (more) negative adverse events during or after training, and (iv) MATT will lead to a more positive perception by participants than the ergometer training.

## Materials and Methods

### Study Design and Procedure

This pilot RCT was conducted at the Godeshoehe, a neurological rehabilitation clinic in Bonn, Germany, with PD patients during their inpatient rehabilitation. Patients' eligibility was assessed by checking medical records and in personal conversation (MG, JS, and JN). If patients were eligible and interested to participate, they were verbally informed about the details of the study (MG, JS, and JN) and received written study information. Written informed consent was obtained from all participants before inclusion in the study. The ethics committee of the medical faculty of the University of Cologne approved the study (reference number 19-1291), which followed the declaration of Helsinki (version 2013, Fortaleza). The trial was registered in the German Clinical Trials Register (DRKS00017687, 25.09.2019) before the first patients' enrollment.

Patients underwent extensive testing before and immediately after the intervention phase to assess cognition, motor function, and patient-centered parameters. Also, we assessed depression, fatigue, quality of life, physical activity level, functional independence in activities of daily living (ADL), freezing of gait, and word fluency 6 weeks later (follow-up) within a telephone interview. The results of cognitive, motor, and patient-centered assessments, as well as the follow-up interviews, will be published elsewhere. All assessors were trained in motor and neuropsychological examinations. The cognitive testing was performed by the neuropsychology staff of the clinic who was not further involved in the study conduction and was blinded for the type of intervention at both time points. The motor function was assessed by two qualified physiotherapists (MG and MK) who were blinded for group allocation in the pre- but not in the post-testing due to limited personnel resources. Patient-centered parameters were assessed with questionnaires that were conducted by trained psychology students (PS, LC, AN, JL, and LE) who were blinded for group allocation at baseline but not after training and in telephone interviews.

After the baseline assessment, patients were randomized into the experimental group (EG) and the control group (CG) by a paper lot that was drawn from a sealed envelope by a person not related to the study. The lots had been prepared before patients' enrollment. Therefore, the numbers 1 to 40 were randomized into the two study conditions (EG and CG) with randomizer.org. Thereafter, paper-lots indicating a number and group allocation were prepared and sealed in an envelope. The lot number (e.g., 00), together with the study ID (MATT_0), served as the participants' ID (e.g., MATT_000) during the study.

**Figure 1:**
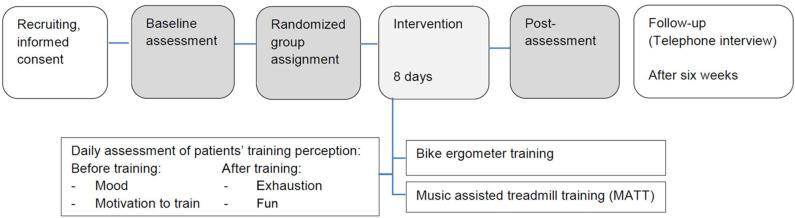
Study protocol.

During the intervention phase, patients received either 20 min of MATT or ergometer training (CG) in addition to the standard rehabilitation program (525 min motor therapy per week plus 210 min cognitive training) for 8 consecutive days excluding weekends and holidays (Figure 1).

Daily, mood and motivation before each training session as well as exhaustion and fun after each training session were evaluated, and training participation was documented.

### Patients and Eligibility Criteria

Participants were inpatients of the neurological rehabilitation center Godeshoehe in Bonn, Germany. They could participate if they had a clinical diagnosis of idiopathic PD [according to UK Brain Bank Criteria; (73)] and confirmed by a neurologist and were non-demented [score ≥17 in the Montreal Cognitive Assessment—MoCA; (74)], had no clinically relevant depression [score ≤10 on the Geriatric Depression Scale—GDS; (75)], or other concurrent neurologic or psychiatric illness, and had no orthopedic or cardiac contraindication to performing the training.

### Interventions

#### Music-Assisted Treadmill Training

The MATT was conducted on Biodex Gait Trainer 3.1 (Biodex Medical System, 20 Ramsay Road, Shirley, NY, USA). This treadmill is an extension of common therapy and training treadmills, as it has an integrated RAC function and a visual feedback option. MATT can be used, for example, to train gait parameters, balance, endurance, and, eventually, also cognition.

For the RAC, the device is equipped with a library of different metronome sounds and music. The selection of music therapy-informed compositions was specially designed by music therapists to expand rhythm-based therapy using spatial, temporal, and force cues (76). Therefore, the beats per minute (bpm) can be adapted to patients walking speed in a range between 45 and 130 bpm. For the visual feedback, a monitor is mounted on the treadmill visualizing patients' steps in comparison with the demanded step. The target step length is visualized by a marked corridor in between two green lines. Patients' alternating steps for right and left foot were shown, and patients were provided with the visual but also verbal feedback (e.g., “good job” or “longer step right/left”). Furthermore, patients received feedback on the step symmetry by the percentage of weight bearing on each foot shown as a bar graph.

During training, the bpm of metronome/music was matched with patients' steps per minute, which were evaluated before the first training serving as an indicator for the baseline walking speed on the treadmill. Walking speed was gradually increased, depending on heartbeat, balance, and synchronization if patients felt comfortable with it. In this study, a metronome (“Metronome Only- E Click Low”) and a piece of music (“Animals Everywhere”) were used. These were found to work best for PD patients in terms of beat recognition and the ability to synchronize in several training sessions before the study onset. Patients started the gait training after walking on the treadmill for 5 min. In this first 5 min, they were provided with real-time visual feedback for their step length, speed, and symmetry. After that, they walked to the metronome for another 5 min, followed by 10 min walking to the music. The visual feedback was provided for the whole training duration. The training was instructed, adapted, supervised, and documented by qualified physiotherapists (MG and MK) and assistants (JS, PS, LC, AN, JL, and LE) who conducted patients' daily ratings before and after training and recorded possible adverse events.

#### Ergometer Training

During the ergometer training, patients trained for 20 min on the ERGO-FIT Cycle 4000 med (ERGO-FIT GmbH & Co. KG, Blocksbergstraße 165, 66955 Pirmasens, Germany). Patients' entry level was at a nominal power of 25 W, which was, if possible, gradually increased over time, depending on medical recommendations, heartbeat, and blood pressure. The ergometer training is a standard endurance treatment at the Godeshoehe rehabilitation clinic. The training was instructed, adapted, supervised, and documented by the team of sports therapists, as this is the usual procedure in the clinic. One person from the study personnel was also around during training, especially to conduct patients' daily ratings before and after training and record possible adverse events.

### Sociodemographic and Clinical Parameters

To allow a description of the study population, sociodemographic (sex, age, education, marital status, and handedness) and clinical parameters (PD duration, DBS yes/no, and DBS duration, physical activity level) were taken from the patients' medical records or assessed in personal communication.

### Outcomes

#### Feasibility of the Study Protocol

The following parameters were used to assess the feasibility of the study protocol: (i) Patients' acceptance of the study protocol was measured as the rate of patients providing their written informed consent to participate in the study out of those patients regarded as eligible for the study; (ii) Transferability of the study interventions into clinical routine was defined by the rate of patients who completed the full study protocol, including the pretesting, the eight training days, and the post-testing (completion and dropout rates).

#### Feasibility of the Music-Assisted Treadmill Training in Comparison With a Treatment as Usual Ergometer Training

To assess the feasibility of the MATT compared with the standard ergometer trainings, the following parameters were assessed: (i) the amount of possible adverse events such as abortions of single training sessions, cardiovascular incidences, falls or almost falls during/after MATT as well as extreme fatigue that caused the cancellation of other trainings after the MATT session or ergometer training were recorded to be compared in the analysis. Therefore, the trainers (MG, MK, JS, PS, AN, LC, JL, and LE) documented the training in a standardized form during training and communicated with the nurses and/or patients about the occurrence of possible adverse events before the next training. (ii) Patients' subjective training perception, including mood, motivation, exhaustion, and fun, was assessed. For each parameter, a four-point Likert scale (e.g., “very happy,” “rather happy,” “rather unhappy,” and “very unhappy”; “not exhausted at all,” “hardly exhausted,” “little exhausted,” and “very exhausted”) was used. Patients in both groups received one questionnaire (asking to rate mood and motivation) before training and the other one (asking to rate exhaustion and fun) after training daily during the intervention phase of the study. Patients were allowed to fill out these questionnaires without the trainers to reduce the risk of bias for responses in favor of the trainers, and they were asked to give their truthful feedback.

#### Cognition

Patients underwent extensive pre- and post-training assessments to evaluate different cognitive domains. The test battery consisted of tests for global cognition [MoCA; (77)], attention [test of attentional performance—TAP; (78), subtests “alertness” and “divided attention”], executive functions (TAP subtest “flexibility” and “Go/NoGo”), working memory [Wechsler Memory Scale, revised version—WMS-R; (79), subtest “digit span reverse” and “block span reverse”], memory (WMS-R subtests “digit span forward” and “block span forward”), and visuocognition [TAP, subtest “visual scanning” and Leistungsprüfsystem—LPS 50+ (80), subtest “mental rotation”]. All tests were performed and evaluated by the team of the neuropsychology department.

#### Motor Function

Motor function was assessed in this analysis with the Hoehn and Yahr Scale (81) and the motor score (part III) of the Movement Disorder Society Unified Parkinson's Disease Rating Scale [UPDRS; (82)] by trained physiotherapists (MG and MK). In the preregistration of the study, we registered further motor outcomes (functional integrity of the lower extremity, walking, balance, gross and fine hand function, and freezing of gait). As this is a preliminary analysis of the ongoing pilot RCT focused on feasibility, only selected outcomes to describe motor impairment were analyzed and presented here.

#### Patient-Centered Outcomes

In terms of patient-centered outcomes, disease severity, depression [GDS; (75)], quality of life [39-item Parkinson's Disease Questionnaire—PDQ-39; (83)], fatigue [16-item Parkinson Fatigue Scale—PFS-16; (84)], and functional independence in ADL [Functional Independence Measure—FIM; (85)] were assessed by psychology students (PS, AN, LC, JL, and LE).

### Statistical Analyses

The statistical analysis was performed with IBM SPSS Statistics 26.0 (Armonk, NY).

An *a priori* sample size calculation or power analysis was not performed, as this is a pilot study especially interested in the feasibility. The planned sample size is 40 patients, 20 in each arm.

For the baseline comparison of demographic data, global cognition, PD related characteristics, and clinical outcomes between the EG and CG, independent sample *t*-tests, Wilcoxon rank-sum tests, or χ2-tests were used as appropriate. Variables and test scores were previously inspected for normal distribution. As the Kolmogorov–Smirnov Test indicated that most variables were not normally distributed, further analyses were conducted with non-parametric tests.

To evaluate the feasibility of the study protocol, the following analyses were conducted: The inclusion rate for all patients was reported. Furthermore, the completion rate and corresponding dropout rate were reported for the EG and CG. To assess reasons for dropout, baseline results of patients following the complete study protocol (completers, C+) and non-completers (C–) were compared by Mann–Whitney *U*-test. To evaluate eventual training-related dropout reasons, the curves in subjective daily measures of C– were visually inspected and descriptively compared with the median curve of C+. To evaluate the feasibility of trainings, reported adverse events are listed for the EG and CG. Moreover, a baseline comparison of C+ patients separated into EG and CG was performed by Mann–Whitney *U*-test. To analyze the course of the subjective training perceptions (motivation, mood, exhaustion, and fun) for the C+ within the EG and CG, Friedman's ANOVA was applied.

The significance level was set at *p* ≤ 0.05; values of *p* ≤ 0.1 were considered as statistical trends. Bonferroni corrections were applied to correct for multiple testing. For the cognition, the adjusted α-level was set at *p* ≤ 0.004 (14 comparisons), for motor function at *p* ≤ 0.025 (two comparisons), and for the patient-centered outcomes at *p* ≤ 0.013 (four comparisons). Corrected *p*-values are reported. Pearson's *r* was calculated as effect sizes and interpreted according to Cohen (86): *r* = 0.10 weak, *r* = 0.30 moderate, *r* = 0.50 strong.

## Results

### Feasibility of the Study Protocol

#### Patients' Acceptance of the Study Protocol

Between September 2019 and March 2020, we checked 54 patients for their eligibility to participate in this pilot RCT. Sixteen patients had to be excluded for not meeting the inclusion criteria; six declined to participate. Finally, 32 patients (24 males; age: 57.56 ± 7.68 years; PD duration: 8.63 ± 6.60 years; 24 DBS patients) were included in this pilot RCT. Therefore, the inclusion rate was 84%.

#### Transferability of Study Protocol Into Clinical Routine

**Figure 2:**
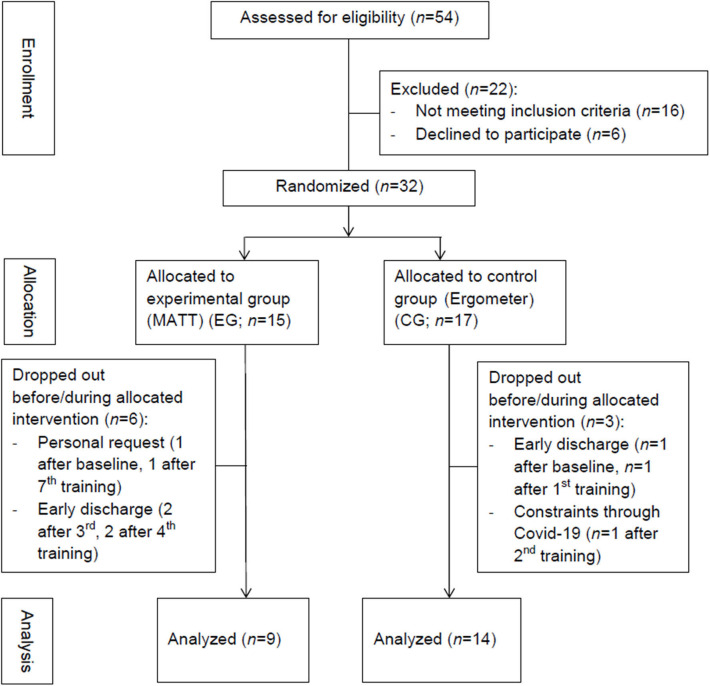
Summary of patients' flow.

During allocation, 15 patients were randomized into the EG and 17 patients into the CG. In the course of the study, nine patients dropped out due to various reasons (EG: *n* = 6; CG: *n* = 3): In the EG, two patients asked to be excluded from the study, one after the baseline assessment and one after the seventh training session. Another four patients had to be discharged from the rehabilitation center before the study protocol was completed (*n* = 2 after the third training, *n* = 2 after the fourth training). Three of these four patients were offered an extended stay but declined. In the CG, two patients were discharged too early as well, one after the baseline assessment and one after the first training. One of the two patients declined an offered extension of rehabilitation. No patient in the CG dropped out for personal reasons. Due to upcoming COVID-19 restrictions, one more patient had to stop after the second training. The completion rate in the EG was 60% and in the CG 82%, with corresponding dropout rates of 40% in the EG and 18% in the CG (Figure 2).

#### Comparison of Completers and Non-completers at Baseline

To further analyze possible dropout reasons, baseline parameters of C+ (*n* = 23, age: 55.83 ± 6.09 years, PD duration: 7.61 ± 4.73 years, 17 DBS patients) and C– (*n* = 9, age: 62 ± 9.77 years, PD duration: 11.23 ± 9.83 years, 7 DBS patients) were examined. Between the C+ and C–, a significant difference in sociodemographic and clinical data was observed for the time since DBS surgery (*U* = 28.000, *p* = 0.041, *r* = 0.42) and the level of activity (*U* = 39.500, *p* = 0.019, *r* = 0.44): time since DBS surgery was longer in C+, and they were physically more active. C+ demonstrated a less motor impairment (Hoehn and Yahr Scale, *U* = 39.500, *p* = 0.005, *r* = 0.49; UPDRS III, *U* = 51.000, *p* = 0.026, *r* = 0.39). Regarding cognition, a significant difference in favor of the C+ was observed for non-verbal short-term memory (WMSR block span forward, *U* = 19.500, *p* = 0.001, *r* = 0.56) as well as statistical trends for non-verbal working memory (WMSR block span reverse, *U* = 41.000, *p* = 0.053*, r* = 0.34) and visual processing accuracy (TAP visual scanning errors, *U* = 43.000, *p* = 0.066, *r* = 0.34). C+ patients showed less depressive symptoms (GDS, *U* = 41.000, *p* = 0.018*, r* = 0.42), tended to experience a higher health-related quality of life (PDQ-39, *U* = 40.500, *p* = 0.050, *r* = 0.36), and higher levels of independence in ADLs (FIM, *U* = 52.000, *p* = 0.070*, r* = 0.33). No other differences between the C+ and C– were identified (Table 1).

**Table 1:** Baseline comparison of completers and non-completers.

		**Completers (C+)**	**Non-completers (C–)**	***U***	**Between-group significance, *p***	**Effect size, *r***
*n*		23	9			
**Sociodemographic and clinical data**						
Sex	Males	19	6	75.500	0.176	
	Females	4	3			
Years of age		55.83 ± 6.09	62.00 ± 9.77	72.500	0.200	
Level of education	No formal education	1	1	75.500	0.134	
	Min. 9 years of education	4	3			
	Min. 12 years of education	5	2			
	12–16 years of education	4	0			
	More than 16 years of education	5	3			
Marital status	Single	2	0	99.000	0.877	
	Relationship	3	0			
	Married	14	9			
	Divorced	4	0			
Handedness (EHI)	Right	21	8	101.000	1.000	
	Left	2	1			
	Bilateral	0	0			
Years since PD diagnosis		7.61 ± 4.73	11.23 ± 9.83	87.500	0.515	
DBS	No	7	2	99.500	1.000	
	Yes	17	7			
Years since DBS surgery		2.24 ± 2.95	0.63 ± 1.48	28.000	0.041^*^	0.42
Freezing of gait (FOGQ)		0.00 (*R =* 21; *n =* 11/23)	14.00 (*R =* 18; *n =* 4/9)	60.000	0.324	
Physical activity level (IPAQ)	Low	5	5	39.500	0.19^*^	0.44
	Moderate	8	3			
	High	9	0			
**Cognition**						
Global cognition (MoCA)^a^		25 (*R =* 10)	25 (*R =* 16)	87.000	0.611	
Attention (TAP)	Alertness (reaction time in ms)^b^	281 (*R =* 822)	300 (*R =* 261)	74.500	0.233	
	Divided attention (omissions)^b^	2 (*R =* 7)	4 (*R =* 14)	70.000	0.161	
Executive functions (TAP)	Flexibility (reaction time in ms)^b^	934 (*R =* 2,458)	1,356 (*R =* 0)	64.000	0.368	
	Flexibility (errors)^b^	2 (*R =* 16)	4 (*R =* 31)	71.500	0.364	
	Inhibition (reaction time in ms)^b^	455 (*R =* 455)	493 (*R =* 299)	76.500	0.268	
	Inhibition (errors)^b^	1 (*R =* 10)	1 (*R =* 14)	93.500	0.678	
Working memory (WMSR)	Digit span reverse^a^	6 (*R =* 7)	5 (*R =* 5)	67.000	0.517	
	Block span reverse^a^	7 (*R =* 8)	4 (*R =* 5)	41.000	0.053^t^	0.34
Memory (WMSR)	Digit span forward^a^	6 (*R =* 8)	6 (*R =* 5)	63.000	0.400	
	Block span forward^a^	8 (*R =* 9)	6 (*R =* 3)	19.500	0.001^*^	0.56
Visuocognition (TAP)	Visual scanning (reaction time in ms)^b^	3,806 (*R =* 5,512)	3,527 (*R =* 8,402)	67.000	0.570	
	Visual scanning (errors)^b^	8 (*R =* 26)	2 (*R =* 8)	43.000	0.066^t^	0.34
Visuocognition (LPS 50+)	Mental rotation^a^	11 (*R =* 25)	10 (*R =* 14)	90.500	0.598	
**Motor function**						
Motor impairment	Hoehn and Yahr^b^	2.5 (*R =* 2.5)	3 (*R =* 1.5)	39.500	0.005^*^	0.49
	UPDRS III^b^	28 (*R =* 50)	43 (*R =* 60)	51.000	0.026^t^	0.39
**Patient-centered outcomes**						
Depressive mood (GDS)^b^		1 (*R =* 9)	4.5 (*R =* 8)	41.000	0.018^t^	0.42
Quality of life (PDQ-39)^b^		31 (*R =* 76)	62 (*R =* 64)	40.500	0.050^t^	0.36
Fatigue (PFS-16)^b^		45 (*R =* 54)	49 (*R =* 36)	69.500	0.322	
ADL independence (FIM)^a^		89 (*R =* 33)	82.5 (*R =* 75)	52.000	0.070^t^	0.33

* *Significant difference*.

a *Higher scores indicate better performance*.

b *Lower scores indicate better performance*.

*P-values are reported for all data, effect sizes only in case of a significant finding. For sociodemographic and clinical data, frequencies, as well as means and standard deviations, are reported. For cognitive, motor, and patient-centered outcomes, medians and ranges are reported. For sociodemographic data, p was set at p ≤ 0.05, whereas p ≤ 0.1 were considered as trends. Corrected p-values are reported for the cognitive data (p ≤ 0.004), the motor function data (p ≤ 0.025), and the patient-centered outcomes (p ≤ 0.013)*.

*t, statistical trend; R, range; EHI, Edinburgh Handedness Scale; FOGQ, Freezing of Gait Questionnaire; IPAQ, International Physical Activity Questionnaire; MoCA, Montreal Cognitive Assessment; TAP, Test of Attentional Performance; WMSR, Wechsler Memory Scale revised; LPS50+, Leistungsprüfsystem; GDS, Geriatric Depression Scale; PDQ-39, 39-item Parkinson's disease questionnaire; PFS-16, 16-item Parkinson Fatigue Scale*.

#### Comparison of Training Perception for Completers and Non-completers

**Figure 3:**
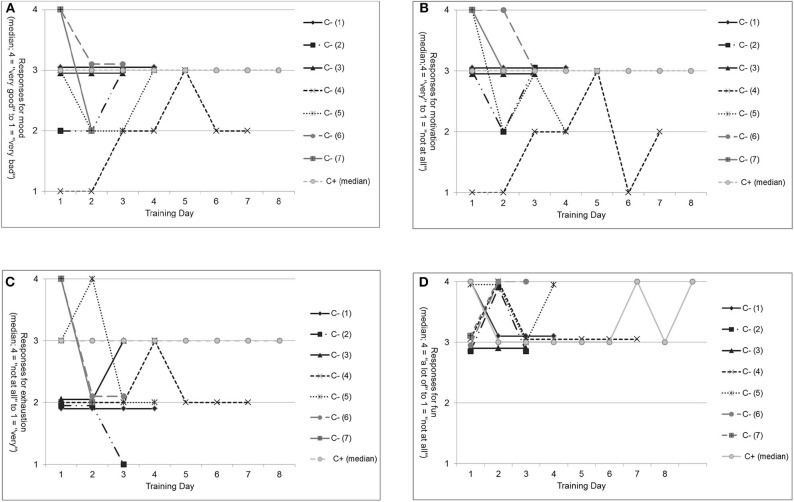
Non-completers' subjective self-perception before and after training compared with the median of completers. **(A)** Mood before each training for non-completers (C– [1] to C– [7]) compared with the median of completers (C+). **(B)** Motivation before each training for non-completers (C– [1] to C– [7]) compared with the median of completers (C+). **(C)** Exhaustion after each training for non-completers (C– [1] to C– [7]) compared with the median of completers (C+). **(D)** Fun after each training for non-completers (C– [1] to C– [7]) compared with the median of completers (C+).

In the C– group, seven of nine patients had trained three to seven times until they dropped out of the study. Therefore, patients' training perceptions before and after the absolved trainings were visualized to explore possible training-induced dropout reasons (Figures 3A–D). Here, some heterogeneity among the seven patients was seen. However, on average, C– patients were in “good mood” and “motivated” before their trainings as well as “a little exhausted” after the training. Furthermore, they had “fun” while training. These results are similar to the median of the C+ group.

Comparing the training perception of those C– patients who trained in the EG (*n* = 5, patient C– [1] to C– [5]) with that in the CG (*n* = 2, patient C– [6] and C– [7]) descriptively, some heterogeneity in patients' reports could be seen. However, most patients in the EG, as well as in the CG, were in “good mood” and “motivated” before training and “exhausted” after the training. Patients in both groups reported that training was “fun.” Only on EG patient (C– [4]) deviated negatively from the others for “mood” and “motivation” but not for “exhaustion” and “fun.”

### Feasibility of the Music-Assisted Treadmill Training and the Ergometer Training

#### Adverse Events of Trainings

In both training groups, no adverse events such as abortions of single training sessions, cardiovascular incidences during or after training, falls or almost falls during or after training, and extreme fatigue that caused the cancellation of other trainings were noted by the patients, trainers, or nurses.

#### Baseline Results for Experimental and Control Groups Without Non-completers

In comparison of sociodemographic and clinical parameters between EG (*n* = 9; age: 56.67 ± 3.61 years, PD duration: 8.64 ± 5.49 years, 5 DBS patients) and CG (*n* = 14, age: 55.29 ± 7.43 years, PD duration: 6.94 ± 4.25, 12 DBS patients), a trend for a statistical difference was noticed, showing that patients in the EG were physically more active (IPAQ, *U* = 34.000, *p* = 0.099, *r* = 0.37). Another trend for a significant group difference was observed for the visual scanning speed (TAP visual scanning, *U* = 31.000, *p* = 0.046, *r* = 0.42), which was faster in the EG compared with the CG. In the EG, quality of life showed a trend to be higher than in the CG (PDQ-39, *U* = 58.500, *p* = 0.087, *r* = 0.29). No further differences were observed between the EG and CG (Table 2).

**Table 2:** Baseline comparison of the experimental and control group.

		**EG**	**CG**	***U***	**Between-group significance, *p***	**Effect size, *r***
*n*		9	14			
**Sociodemographic and clinical data**						
Sex	Males	8	11	56.500	0.630	
	Females	1	3			
Years of age		56.67 ± 3.61	55.29 ± 7.43	54.500	0.609	
Level of education	No formal education	0	1	53.000	0.952	
	Min. 9 years of education	1	3			
	Min. 12 years of education	2	3			
	12–16 years of education	1	0			
	More than 16 years of education	5	7			
Marital status	Single	1	1	57.000	0.643	
	Relationship	1	2			
	Married	6	8			
	Divorced	1	3			
Handedness (EHI)	Right	9	12	54.000	0.502	
	Left	0	2			
	Bilateral	0	0			
Years since PD diagnosis		8.64 ± 5.49	6.94 ± 4.25	52.000	0.504	
DBS	No	4	2	44.000	0.162	
	Yes	5	12			
Years since DBS surgery		1.62 ± 2.55	2.50 ± 3.17	24.500	0.583	
Freezing of gait (FOGQ)		5 (*R =* 16; *n =* 5/9)	0 (*R =* 21; *n =* 6/14)	54.000	0.559	
Physical activity level (IPAQ)	Low	0	5	34.000	0.099	0.37
	Moderate	4	4			
	High	5	4			
**Cognition**						
Global cognition (MoCA)^a^		25 (*R =* 5)	25 (*R =* 10)	47.500	0.471	
Attention (TAP)	Alertness (reaction time in ms)^b^	261 (*R =* 257)	288.5 (*R =* 822)	58.500	0.793	
	Divided attention (omissions)^b^	1 (*R =* 6)	2.5 (*R =* 7)	46.500	0.306	
Executive functions (TAP)	Flexibility (reaction time in ms)^b^	1,067 (*R =* 1,429)	834 (*R =* 2,458)	48.000	0.369	
	Flexibility (errors)^b^	2 (*R =* 15)	3.5 (*R =* 16)	59.000	0.815	
	Inhibition (reaction time in ms)^b^	473 (*R =* 281)	425.5 (*R =* 225)	38.000	0.124	
	Inhibition (errors)^b^	1 (*R =* 4)	1 (*R =* 10)	59.000	0.813	
Working memory (WMSR)	Digit span reverse^a^	6 (*R =* 7)	6 (*R =* 3)	46.000	0.274	
	Block span reverse^a^	6 (*R =* 8)	7.5 (*R =* 7)	44.000	0.228	
Memory (WMSR)	Digit span forward^a^	6 (*R =* 5)	6.5 (*R =* 6)	55.500	0.645	
	Block span forward^a^	8 (*R =* 7)	7.5 (*R =* 6)	46.000	0.294	
Visuocognition (TAP)	Visual scanning (reaction time in ms)^b^	3,409 (*R =* 1,987)	4,031 (*R =* 5,021)	31.000	0.046^t^	0.42
	Visual scanning (errors)^b^	5 (*R =* 16)	8.5 (*R =* 24)	45.000	0.267	
Visuocognition (LPS 50+)	Mental rotation^a^	9 (*R =* 25)	12.5 (*R =* 12)	51.000	0.465	
**Motor function**						
Motor impairment	Hoehn and Yahr^b^	2.5 (*R =* 1.5)	2.5 (*R =* 2.5)	62.500	0.981	
	UPDRS III^b^	28 (*R =* 35)	28 (*R =* 42)	62.000	0.963	
**Patient-centered outcomes**						
Depressive mood (GDS)^b^		2 (*R =* 8)	1 (*R =* 7)	41.500	0.172	
Quality of life (PDQ-39)^b^		30 (*R =* 72)	36 (*R =* 76)	58.500	0.087^t^	0.29
Fatigue (PFS-16)^b^		45 (*R =* 45)	44.5 (*R =* 54)	61.000	0.914	
ADL independence (FIM)^a^		89 (*R =* 14)	88.5 (*R =* 33)	56.000	0.672	

a *Higher scores indicate better performance*.

b *Lower scores indicate better performance*.

*P-values are reported for all data, effect sizes only in case of a significant finding. For sociodemographic and clinical data, frequencies, as well as means and standard deviations, are reported. For cognitive, motor, and patient-centered outcomes, medians and ranges are reported. For sociodemographic data, p was set at p ≤ 0.05, whereas p ≤ 0.1 were considered as trends. Corrected p-values are reported for the cognitive data (p ≤ 0.004), the motor function data (p ≤ 0.025), and the patient-centered outcomes (p ≤ 0.013)*.

*t, statistical trend; R, range; EHI, Edinburgh Handedness Scale; FOGQ, Freezing of Gait Questionnaire; IPAQ, International Physical Activity Questionnaire; MoCA, Montreal Cognitive Assessment; TAP, Test of attentional Performance; WMSR, Wechsler Memory Scale revised; LPS50+, Leistungsprüfsystem; GDS, Geriatric Depression Scale; PDQ-39, 39-item Parkinson's disease questionnaire; PFS-16, 16-item Parkinson Fatigue Scale*.

#### Patients' Training Perception for Experimental and Control Groups Without Non-completers

**Figure 4:**
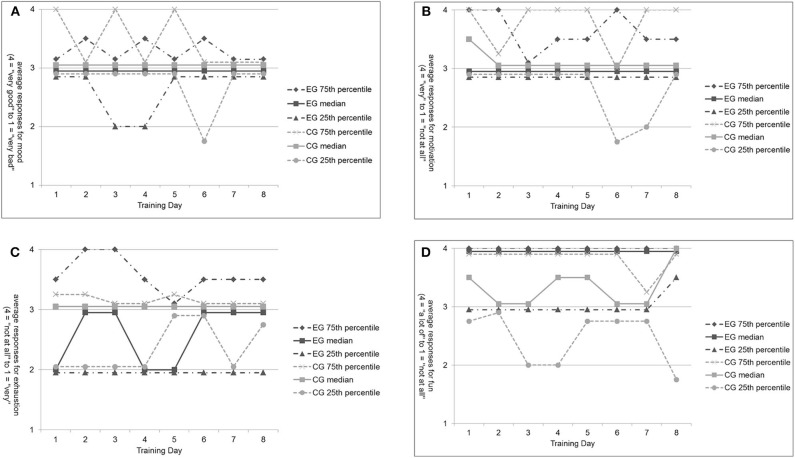
Patients' subjective self-perception before and after training for the experimental and control group without non-completers. **(A)** Mood before each training for EG and CG patients. **(B)** Motivation before each training for EG and CG patients. **(C)** Exhaustion after each training for EG and CG patients. **(D)** Fun after each training for EG and CG patients.

Comparing these parameters for the C+ separately for the EG and the CG for all trainings (Figures 4A–D), it was observed that patients in the EG were in “good mood” and “motivated” before training but showed some variance in post-training exhaustion over the eight trainings ranging from “a little exhausted” to “exhausted.” Furthermore, patients in the EG reported having “a lot of fun” during training. No significant changes over time within the EG were identified. In the CG, patients were also in “good mood” and mostly “motivated” before training. However, patients were significantly less motivated to train on the sixth day [χ^2^(2)= 18.293, *p* = 0.011] compared with those on all other training days. Patients in the CG were “a little exhausted” after training, and their report of fun during training varied between “fun” and “a lot of fun.” In the CG, no further changes were observed.

## Discussion

In the present feasibility analysis, we aimed to evaluate the feasibility of the study protocol in terms of patients' acceptance of the study and the transferability into clinical routines as well as the feasibility of training defined by the occurrence of adverse events and patients' training perception. The main findings were that the study protocol was well-accepted by PD patients with and without DBS. However, the high dropout rate, especially in the EG, indicated the transferability of the study protocol only. Nevertheless, the analysis of the dropout cohort did not reveal intervention-related dropout reasons (but patient-related differences between C+ and C–). Furthermore, the MATT, as well as the standard ergometer training, showed no adverse events and was positively perceived by PD patients with and without DBS.

### Feasibility of the Study Protocol

#### Patients' Acceptance of the Study Protocol

In this feasibility analysis, patients' acceptance of the protocol was measured as the rate of participating patients out of eligible patients (=inclusion rate), which was 84%. In a comparable study by Calabrò et al. (67), also examined the effects of MATT, an inclusion rate of 94% was obtained. It has to be noted that in the present study, six eligible patients declined to participate. However, it is difficult to give reasons for the rejection, as patients were allowed to quit without giving reasons. Nevertheless, it would be informative to find inclusion rates in studies more often.

#### Transferability of Study Protocol Into Clinical Routine

The transferability of the study protocol into clinical routines was evaluated by the completion and dropout rate. The present feasibility analysis revealed, a completion rate of 60% (and corresponding 40% dropout) in the EG, as well as 82% completion (and corresponding 18% dropout) in the CG. As previous studies on MATT (64, *n* = 40; 67, *n* = 50) did not observe any dropouts, we did not anticipate such a high dropout-rate for our study. Thus, an accordingly high dropout rate needs to be considered when calculating sample sizes for future (MATT-) studies in an inpatient rehabilitation setting. Furthermore, it might be relevant to evaluate if process optimization in the context of study planning might decrease dropout-rates. This might include communicating with patients in advance if they would be interested in an extended length of stay if offered. However, this would be irrelevant for patients' who are discharged earlier due to resolved medical indication. In these cases only shorter training protocols would help.

#### Comparison of Completers and Non-completers at Baseline

To elucidate the high dropout rate from our study, the group of C– was closely examined. This analysis revealed that C+ patients were in better health constitution than C– patients. It was especially striking that C– patients had received their DBS stimulation (DBS: *n* = 7/ 9) after a longer disease duration than C+ (DBS: *n* = 17/24) and that C– patients were less physically active than C+ patients. There are several ways to explain the observed differences.

One possibility is that due to shorter disease duration and younger age, patients in the C+ could be more physically active, as they were less impaired, which had a positive impact on cognition, motor functioning, and patient-centered outcomes, whereas patients in the C– group, due to longer disease duration and older age, had adopted a more sedentary lifestyle (15). Inactivity, according to van Nimwegen et al. (15), is related to disease severity, motor impairment, and greater disability in ADL. Today, it is common sense that physical activity is an essential tool to prevent age-related (87–89) and disease-related decline (90–92). It is, therefore, plausible that these observed baseline differences resulted in less ambition to complete the study protocol. For future studies, it might, therefore, be recommendable to stratify concerning these parameters during randomization.

Another explanation might be that, due to higher levels of health-related quality of life, ADL independence, and less depressive symptoms, C+ patients faced fewer barriers to be physically active, which resulted in better health conditions, whereas C– patients experienced the opposite. It is well-known that mood, self-perception, the stage of the disease, and the faith in being able to modify disease progression influence the motivation and activity levels (11, 45, 93–95). It can, therefore, be hypothesized that C– patients had lost their self-efficacy expectancy and, therefore, did not consider themselves as capable of modifying disease progression through training.

As the majority of C+ (71%) and C– patients (78%) had a DBS, the time point of DBS surgery to disease duration might also play an important role, as patients who received their DBS at an earlier time point of the disease might benefit from the early stimulation concerning motor functions and ADL independence as well as the quality of life and mood (96, 97). However, the effects of DSB on cognition are discussed controversy (26, 96). Again, reports of dropout cohorts might help to gain further insight into the question if early stimulated patients show higher rates of therapy adherence, which could be a relevant argument for DBS.

However, it is also possible that the time since surgery to study inclusion was crucial for the observed differences in C+ and C– (time since surgery in CG: 0.63 ± 1.48 years; in EG: 2.24 ± 2.95 years). C– patients were more recently stimulated than C+ patients, which might have caused that C– patients relied more on the adaptation of the DBS settings and medication by the neurologist instead of recognizing their own responsibility to train as an important modifier of disease progression. Furthermore, the short duration since DBS surgery might have caused that patients were less motivated to stay in the rehabilitation clinic, as they were usually referred directly from the acute clinic resulting in a long absence from home. Studies evaluating these aspects of therapy adherence, especially in PD DBS, are needed to schedule rehabilitation more effectively and to increase patients' education on these aspects.

However, from the results obtained in the present feasibility analysis, we can only speculate on causality. Nevertheless, it gives an interesting first insight into a group of C–, which could be extended only by more trials examining their dropout cohorts. This might be especially valuable to answer the question of why our therapy aims do not reach the patient and when therapy should start in the course of disease progression. This information would also extend the knowledge on training adherence of PD patients, especially with DBS.

#### Comparison of Training Perception for Completers and Non-completers

The visualized reports of self-perception before and after training for those C– patients who absolve trainings (*n* = 7) did not support the general assumption that withdrawal from a study might be related to the subjective experience of the study intervention. This observation is conclusive, as only one (C– [4]) of these seven patients left the study on personal request. However, another three of these patients were offered an extension of stay, which they declined. On the one hand, the patient who quit the study after the seventh training for personal reasons reported low mood and motivation before most training sessions. On the other hand, he reported having fun during the MATT. Therefore, it can be assumed that the withdrawal was more related to a general factor rather than the training itself. One possible factor could be resignation due to loss of self-efficacy, which was shown to be a predictor of physical activity in PD patients (15, 93–95). Another possible explanation might be impaired self-awareness of motor and cognitive functions (anosognosia), which has been reported in PD patients (98). Although it is more likely to appear in more cognitively impaired or depressed patients (99), it was found that some PD patients with and without DBS were unable to adequately judge their cognitive and motor skills (99, 100). Patients, who judge their abilities incorrectly, might not recognize the need for training and therefore care less about it. These phenomena might also explain the second withdrawal upon personal request by another patient in the EG, who left the study before the first training. However, the influences of these two factors are still underinvestigated concerning training participation/adherence. Here, again, the analysis of dropout cohorts could be informative.

### Feasibility of the Music-Assisted Treadmill Training and the Ergometer Training

#### Adverse Events of Training

The additional 20-min MATT, as well as the ergometer training, was feasible for all patients in this study, and no adverse events were reported. This observation extends especially the limited evidence on the safety training approaches for PD DBS patients (72).

#### Baseline Results for Experimental and Control Groups Without Non-completers

Although some heterogeneity was observed between the EG and CG, both groups were mostly comparable at baseline. The statistical trends for more physical activity, better visual scanning speed, and higher quality of life in favor of the EG might result from different group sizes and need to be confirmed with the final sample size. However, if these differences are found to be robust, they need to be considered when interpretation post-training results.

#### Patients' Training Perception for Experimental and Control Groups Without Non-completers

On average, patients were in “good mood” and “motivated” before training independent of the group allocation, although some variance was noticed within each group. These results are conclusive, as patients in both groups did not show depressive symptoms that could have influenced mood more generally, and patients volunteered to participate in the study, probably representing motivation. Furthermore, it shows that patients' moods and motivations were obviously not dependent on the form of training. It is possible that patients' perception before training was influenced by the knowledge that they participated in a study and the closer relationship to the constant study team compared with more frequently changing therapists during other treatments. This observation is in line with findings from other studies, showing that the trainer (exercise leader) is of crucial importance for patients' motivation and adherence to training as well as social interaction during training (94, 95).

The reports of “exhaustion” varied more in the EG than in the CG after training. The form of training might explain the greater variance within the EG. The ergometer training in the CG is as an aerobic, seated, single-task training, which is probably less demanding for PD patients than the aerobic, upright, dual-task MATT in the EG. However, after the fifth training, patients in the EG seemed to have gotten more used to the training, resulting in less exhaustion for the remaining days of training. Furthermore, patients' reports of fun during training differed descriptively between the two groups. Patients in the EG had, on the average, “a lot of fun” during MATT, whereas patients in the CG had “fun.” The MATT seems to be a little more enjoyable than the ergometer training, which is eventually an effect of the musically assisted training. The power of music to ease training is a common experience (101), which was proven to be relevant for neurological rehabilitation (57, 63, 64, 67–69, 76, 102–104). It might also be an effect of the novelty of the approach. However, this result is of high importance regarding patient reported outcome measurements, and it can be regarded as an important indicator for patients' therapy adherence.

However, patients were not asked in detail about the reasons for their mood and motivational status as well as the exhaustion and what had caused the experienced fun. Only a more elaborated questionnaire or a semi-structured interview might reveal further insights here. This information will be especially needed when planning and conducting even more patient-centered therapies and should be implemented in future feasibility trials.

### Strength and Limitations

In Germany, inpatient rehabilitation is a common setting for many PD patients with and without DBS. Therefore, it is especially important to know if newly provided treatments are accepted by patients, feasible in the clinical routines, and safe for the patient clientele. In this regard, it is important to adapt the study protocols to the settings and circumstances of therapy, rather than to adapt the settings to the preferred study protocol to gain transferable insights. It is a clear strength of the study protocol to be designed for a realistic clinical setting. Thus, we used a training protocol that was able to schedule in the training plans of the rehabilitation clinic, and we avoided high-intensity training like it was used in user studies using MATT (67–69). Furthermore, we covered a realistic sample of PD patients in a rehabilitation clinic, including those with DBS, as well as patients who underwent adjustment of medication and DBS settings. However, it should be kept in mind that our results cannot be generalized to the global PD population, and caution should be expressed regarding transferability.

Furthermore, in this clinical setting, unforeseen, system-related changes, like early discharges, can occur. Moreover, the diversity of rehabilitation treatments combined with a large amount of time required for therapy makes it more challenging to differentiate intervention-induced effects from general improvements. Therefore, controlled designs are essential. As already mentioned, we aimed to implement the MATT in a realistic setting. Thus, we did not choose a treadmill training without music assistance as a CG but ergometer training, which is the standard endurance training provided by sports therapists in the rehabilitation clinic Godeshoehe e.V., Bonn. As a limitation, this design does not allow identifying the specific effect of music on the patients' feasibility; future studies should, therefore, consider a CG receiving treadmill training without music.

Furthermore, the study represents a realistic though a small cohort of PD patients with and without DBS during their inpatient rehabilitation. Consequently, the stability of medication and DBS settings was none of our inclusion criteria. This probably has some effects on the interpretability of present and future results and should be avoided when further assessing the behavioral effects of MATT.

Although dropout rates were high, especially in the EG, the in-depth analysis of the dropout cohort is a strength of this feasibility analysis and did not reveal training-induced dropout reasons. However, high dropout rates will limit the interpretability of behavioral results in future analyses of our data. Furthermore, due to ethical aspects, we were not allowed to ask for explicit reasons for dropout, and patients were allowed to quit without giving reasons. Thus, interpretations in this respect remain speculative. Notably, dropouts only occurred in patients during the recruitment phase as well as in one patient during the intervention period.

This feasibility analysis showed that the MATT did not cause any negative adverse events in PD patients with and without DBS and seemed to be a safe training during the rehabilitation. Nevertheless, further studies with larger sample sizes investigating the feasibility of MATT, especially in PD DBS patients, are needed.

The fact that the feasibility of trainings was not only assessed by the reported adverse events but also by patients' subjective perception of training is another strength of the study. Nevertheless, the applied Likert scales provide no understanding of the reasons for the chosen answers. Future evaluations of patients' training perception would, therefore, benefit from more extensive questionnaires or a mixed-methods approach, including qualitative interviews. More detailed feedback might also help to identify eventual between-group differences.

The present feasibility analysis included a detailed sample description based on results from an extensive cognitive, motor, and patient-centered test battery. All raters in the study were blinded during pretraining assessments. Nevertheless, it was a clear limitation that raters of motor function and patient-centered parameters were not blinded in the post-assessment, and raters of patients' daily training perception were not blinded due to lack of personnel resources. However, raters of cognition were blinded in pre- and post-assessments, as the staff of the neuropsychology department performed these tests. Future studies should provide complete single blinding of all raters at all time points.

We set an alpha level of ≤0.1 as a statistical trend, which can be discussed as too generous. However, we also focused on effect sizes to get a deeper understanding of whether differences between the groups and time points of measurement occurred.

## Conclusion

MATT was shown to be a feasible, safe, and enjoyable training for PD patients with and without DBS, although RCTs with larger sample size are needed to examine the training efficacy and to make further recommendations for training possibilities in PD patients with and without DBS. Furthermore, the dropout cohort analysis revealed some exciting first insights into possible dropout reasons that go beyond the form of intervention. Therefore, research would benefit from a common practice of dropout analyses, as this would enhance our understanding of patients' therapy adherence and expectations. Finally, MATT should also be used in other settings to examine the generalizability of the findings for the general PD population.

## Data Availability Statement

The datasets presented in this article are not readily available because currently, we are working on a further publication. Afterwards, the data related to this study can be accessed on request from the corresponding author (Elke Kalbe). Requests to access the datasets should be directed to elke.kalbe@uk-koeln.de.

## Ethics Statement

The studies involving human participants were reviewed and approved by Ethics Committee of the Medical Faculty of the University of Cologne (reference number 19-1291). The patients/participants provided their written informed consent to participate in this study. This clinical trial is registered with the German Clinical Trials Register, under the following trial registration number: DRKS00017687.

## Author Contributions

MG: conception of the study, data acquisition, data analysis, data interpretation, and drafting the manuscript. JS and A-KF: conception of the study, data acquisition, data interpretation, and revising the manuscript. JN: conception of the study, data acquisition, data analysis, data interpretation, and revising the manuscript. JZ: conception of the study and data acquisition. PS, AN, LC, JL, LE, and MK: data acquisition. AO: data analysis and revising the manuscript. NA and HK: conception of the study and revising the manuscript. EK: conception of the study, data analysis, data interpretation, and revising the manuscript. All authors contributed to the article and approved the submitted version.

## Conflict of Interest

MG has received honoraria from Bernafon AG, Bern, Switzerland. A-KF has received a grant from the German Parkinson Society and honoraria from ProLog Wissen GmbH, Cologne, Germany, and pro audito Switzerland, Zürich, Switzerland. A-KF is the author of the cognitive training program NEUROvitalis but receives no corresponding honoraria. NA has received honoraria from Medtronic for lecturing and consulting and from Boston Scientific and Merz Pharmaceuticals for lecturing. EK has received grants from the German Ministry of Education and Research, ParkinsonFonds Deutschland gGmbH, and the German Parkinson Society and honoraria from Oticon GmbH, Hamburg, Germany; Lilly Pharma GmbH, Bad Homburg, Germany; Bernafon AG, Bern, Switzerland; and Desitin GmbH, Hamburg, Germany. EK is author of the cognitive training program NEUROvitalis but receives no corresponding honoraria. The remaining authors declare that the research was conducted in the absence of any commercial or financial relationships that could be construed as a potential conflict of interest.
